# Dependence structure analysis of multisite river inflow data using vine copula-CEEMDAN based hybrid model

**DOI:** 10.7717/peerj.10285

**Published:** 2020-11-06

**Authors:** Hafiza Mamona Nazir, Ijaz Hussain, Muhammad Faisal, Alaa Mohamd Shoukry, Mohammed Abdel Wahab Sharkawy, Fares Fawzi Al-Deek, Muhammad Ismail

**Affiliations:** 1 Department of Statistics, Quaid-i-Azam University, Islamabad, Pakistan; 2 Faculty of Health Studies, University of Bradford, Bradford, UK; 3 Bradford Institute for Health Research, Bradford Teaching Hospitals NHS Foundation Trust, Bradford, UK; 4 Arriyadh Community College, King Saud University, Riyadh, Saudi Arabia; 5 KSA workers University, Nsar, Egypt; 6 Department of Statistics, COMSATS University Islamabad, Lahore Campus, Lahore, Pakistan

**Keywords:** Canonical-vine, Pair copula construction, Complete ensembe empirical mode decomposition with adaptive noises

## Abstract

Several data-driven and hybrid models are univariate and not considered the dependance structure of multivariate random variables, especially the multi-site river inflow data, which requires the joint distribution of the same river basin system. In this paper, we proposed a Complete Ensemble Empirical Mode Decomposition with Adaptive Noise (CEEMDAN) Vine copula-based approach to address this issue. The proposed hybrid model comprised on two stages: In the first stage, the CEEMDAN is used to extract the high dimensional multi-scale features. Further, the multiple models are used to predict multi-scale components and residuals. In the second stage, the residuals obtained from the first stage are used to model the joint uncertainty of multi-site river inflow data by using Canonical Vine. For the application of the proposed two-step architecture, daily river inflow data of the Indus River Basin is used. The proposed two-stage methodology is compared with only the first stage proposed model, Vector Autoregressive and copula-based Autoregressive Integrated Moving Average models. The four evaluation measures, that is, Mean Absolute Relative Error (MARE), Mean Absolute Deviation (MAD), Nash-Sutcliffe Efficiency (NSE) and Mean Square Error (MSE), are used to observe the prediction performance. The results demonstrated that the proposed model outperforms significantly with minimum MARE, MAD, NSE, and MSE for two case studies having significant joint dependance. Therefore, it is concluded that the prediction can be improved by appropriately modeling the dependance structure of the multi-site river inflow data.

## Introduction

In the past few decades, reliable prediction of rivers inflow has gained popularity in all water-related departments because of their crucial role in the reservoir, irrigation management, water planning, risk evaluation and flood controlling (Porporato & Ridolfi, 2001; Jandhyala, Liu & Fotopoulos, 2009; Di, Yang & Wang, 2014; Tiwari et al., 2017; Nazir et al., 2019). Johnston & Smakhtin (2014) reviewed the importance of river data and concluded that river inflow data is an indispensable component of water resources. For many rivers and water storage systems, a joint prediction of inflow at multi-site, which not only accounts for the inflow characteristics of individual streams but also consider their intersite correlations, is necessary for planning water resources and flood control (Wang & Robertson, 2011). Several single-site and multi-site models have been developed to predict river inflow (Sharma & O’Neill, 2002; Wu & Chau, 2010; Wang & Robertson, 2011; Oyebode, Otieno & Adeyemo, 2014; Devia, Ganasri & Dwarakish, 2015; Wang et al., 2018). Among single-site models, traditional statistical models that include Autoregressive (AR), Moving Averages (MA), Autoregressive Integrated Moving Average (ARIMA), Autoregressive Conditional Heteroscedasticity (ARCH) and Generalized ARCH (GARCH) have been efficiently used to predict river inflow data (Ghimire, 2017). However, these are commonly used methods but have some drawbacks that these are unable to capture non-linear, non-stationary, and inter-dependence characteristics of time-series data such as river inflow data (Box, 1970; Sharma & O’Neill, 2002; Wu & Chau, 2010; Oyebode, Otieno & Adeyemo, 2014; Devia, Ganasri & Dwarakish, 2015; Wang et al., 2018). The limitations of such non-stationary and non-linearity led to the emergence of a new paradigm named as data-driven or intelligence models (Wu & Chau, 2010; Oyebode, Otieno & Adeyemo, 2014; Das & Ghosh, 2017).

Several data-driven approaches have been recognized as useful tools to model complex non-stationary and non-linear river inflow data. For example, *K*-Nearest Neighbors, model tree (Oyebode, Otieno & Adeyemo, 2014), computational intelligence (Das & Ghosh, 2017), Genetic Algorithm, Support Vector Machine, Neural Networks (NN) includes Artificial Neural Network (ANN) and Artificial Intelligence (Tiwari et al., 2017). These data-driven models can learn complex behavior, which is an inherent part of river inflow data, without considering any assumption about data. El-Shafie, Taha & Noureldin (2007) discussed that river inflow forecasting is an essential procedure for proper water operation. They proposed an adaptive-neuro fuzzy inference system to forecast the monthly inflow data. Wu & Chau (2010) explained in their review that the data-driven approaches performed better than the traditional statistical models to predict the non-linear data. However, data-driven models may suffer an overfitting problem and are sensitive to parameter selection (Nazir et al., 2019). Moreover, data-driven models ignored the time-varying or multi-scale characteristics of time series data. Several hybridization methods have been proposed to extract multi-scale or time-varying information from time-series data. These methods can be combined data-driven models with some data-preprocessing data methods, that is, Wavelet Analysis (WA), Empirical Mode Decomposition (EMD), Ensemble EMD (EEMD) and Complete Ensemble Empirical Mode Decomposition with Adaptive Noise (CEEMDAN) (Ji, Lu & Tang, 2012; Karthikeyan & Kumar, 2013; Kang et al., 2017). The extracted multi-scale information is further used as input in data-driven models to efficiently predict complicated time-series data (Di, Yang & Wang, 2014; Panigrahi & Behera, 2017). Quality of prediction can be improved by independent modeling of these multi-scale components when these multi-scale components were modeled independently (Karthikeyan & Kumar, 2013; Nazir et al., 2019). Di, Yang & Wang (2014) introduced a four-stage hybrid model combining EMD/EEMD with the radial basis function of NN methods. They found that their hybrid model performs better than the conventional single time series models. Nazir et al. (2019) also proposed a hybrid model comprised of WA/EMD-CEEMDAN as a data pre-processing technique to model the inflow data.

However, all traditional statistical, data-driven and hybrid models are only useful to deal with non-linear, non-stationary, and multi-scale data. They did not model the dependance structure of the multi-site rivers inflow data, which requires the joint distribution of the same river basin system. Medda & Bhar (2019) deals with the comparison of single-site and multi-site streamflow prediction models. Their study revealed that the cross-correlation between multi-site rivers enhances the performance of streamflow predictions. Therefore, failure to incorporate such multi-site dependance in predicting rivers inflow may produce an inaccurate and unreliable prediction. Several models have also been developed for modeling multi-site rivers inflow data (Ledolter, 1978; Porporato & Ridolfi, 2001; Sharma & O’Neill, 2002; Jandhyala, Liu & Fotopoulos, 2009; Hao & Singh, 2013; Aghakouchak, 2014; Aranda & García-Bartual, 2018). Jandhyala, Liu & Fotopoulos (2009) proposed a Gaussian formulation to detect changes in mean rivers flow by considering the six rivers dependance structure which flows in the same region. They reached on the conclusion that if the assumption of temporal independence is satisfied, their proposed multivariate framework performs reasonably well in predicting average rivers flow.

Dependence between sites can be modeled by copulas (Min & Czado, 2010; Aghakouchak, 2014; Liu et al., 2015; Balistrocchi et al., 2017; Aranda & García-Bartual, 2018; Addo, Chanda & Metcalfe, 2018; Wang et al., 2018). Hao & Singh (2013) have proposed the maximum entropy copula for multi-site streamflow simulation and shown a reasonable agreement with observed streamflow. Aranda & García-Bartual (2018) used the copula theory for the probabilistic modeling of rivers flow. However, the use of Copula is restricted to bivariate dependance only. For modeling the high dimensional data, vines including Regular Vine (R-Vine), Canonical Vine (C-Vine) and Drawable Vine (D-Vine) have been introduced (Czado & Aas, 2013; Liu et al., 2015; Bedford & Cooke, 2001; Bedford, Daneshkhah & Wilson, 2016). Vines are used for building a high-dimensional probabilistic dependance structure, which may be comprised on the product of simple bivariate and conditional bivariate distribution functions. Bedford, Daneshkhah & Wilson (2016) approximated the complex joint uncertainty by using vines due to its flexibility of constructing high-dimensional multivariate distributions in the hierarchy, which proves better than the other simple bivariate elliptical and Archimedean copulas. However, copula/vines have an underlying assumption that the time series data or a variable should be linear and stationary, which means there is no serial correlation between a time series variable and its lagged version, which usually does not meet in complex river inflow data (Sklar, 1959; Czado et al., 2009; Lee & Salas, 2011; Yusof, Kane & Yusop, 2013). Laux et al. (2011) proposed a hybrid model based on ARMA-GARCH to deal with a linearity and stationery: Independent Identically Distributed (IID) requirement of the Copula. They first transformed the non-linearity of complex data using the ARMA-GARCH model, then the filtered residuals resulting from ARMA-GARCH are used as input in Copula. They concluded that IID transformations through ARMA-GARCH are imperative before modeling multivariate dependance structure between hydrological data to avoid bias induced due to volatile dependance. Singh et al. (2018) also developed a hybrid copula-based model for seasonal data. They incorporated ARMA with Copula to effectively filter out the inter-dependence, which are proved fruitful in accurately simulating pre and post-monsoon data. However, for multi-site river inflow prediction, the inflow at individual river sites and dependance structure among different locations in the same river basin system, which involves nearby catchment characteristics of rivers, should preserve all statistical properties.

In summary, several data-driven and hybrid models have been developed which are univariate and do not recognize the dependency structure of multivariate random variables, particularly the multi-site river inflow data, which needs the joint dissemination of the same river basin system (Nazir et al., 2019; Bedford, Daneshkhah & Wilson, 2016). In this study, we proposed two separate approaches CEEMDAN and C-Vine, to consider the dependance structure of multivariate random variables, especially the multi-site river inflow data, while preserving all statistical properties: non-linear, non-stationary, and multi-scale.

## Proposed Method

To predict the multi-site rivers daily inflow data, a copula-based CEEMDAN hybrid model is proposed which works in two-stages to model the multi-scale and mutual dependance of multi-site streams inflow as follows:

*In the first stage*: to get the independent residuals (which are used in the second stage to model the joint uncertainty); first, there was a need to model the multi-scale components of time series data more appropriately. The CEEMDAN decomposition method is applied to extract high dimensional multi-scale elements (Intrinsic Mode Function (IMF)) from each river’s inflow data separately. The dimensionality of these IMFs is reduced (CEEMDAN-R) by adding the respective high and low multi-scale IMFs, respectively, except the first two IMFs and the last residual. Next, the Multiple Models (MM), that is, Group Method of Data Handling type Neural Network (GMDH-NN) and the Revised form of GMDH type Neural Network (RGMDH-NN) and ARIMA are selected to predict the IMFs and residuals (CEEMDAN-R-MM) (Nazir et al., 2019). The residuals of this stage are further used as input to model the mutual dependance of multi-site rivers inflow.

**Figure 1:**
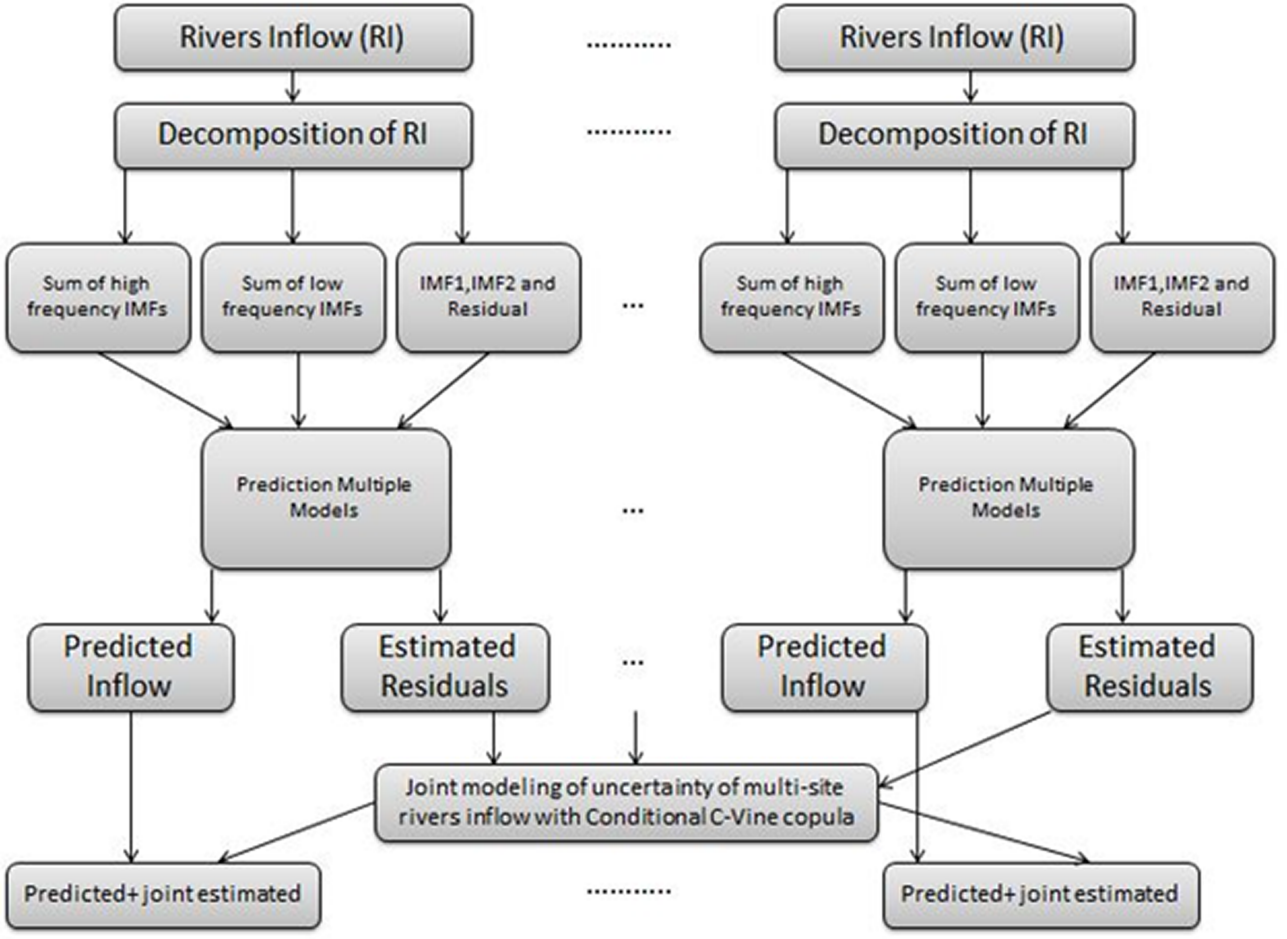
The proposed C-Vine based CEEMDAN-R-MM structure to predict multi-site hydrological time series data.

*In the second stage*: a C-Vine copula function is used to model mutual dependance of multi-site rivers inflow of the same river basin system. The C-Vine is selected due to its functionality of selected a root node, which enhances the sum of pairwise dependance to this node. The schematic view of the proposed model is given in Fig. 1. For convenience, the proposed method is denoted as C-Vine based CEEMDAN-R-MM method. The detail description of the proposed method is described as follows:

### The CEEMDAN as a decomposition method

In this study, the CEEMDAN method introduced by Torres et al. (2011) is used to decompose the daily rivers inflow time series data, which is briefly described as follows:

1. The CEEMDAN add white Gaussian noises on the lines of EEMD, in original inflow data as follows:

(1) $$y\left( t \right) = x\left( t \right) + {w_0}{n^j}\left( t \right)$$

where $(j = 1,2, \ldots ,m$) *m*th ensemble, $x\left( t \right)$ is original inflow data and ${w_0}$ is the white noise amplitude. The first IMF is found in the usual way as in EEMD defined as:

(2) $$\widetilde {\rm IM{F_1}} = \mathop \sum \limits_{j\,=\,1}^m \displaystyle{{\rm IMF_{{\it j}1}^{\it m}} \over m}$$

2. Compute the remainder of original inflow from the first IMF through the following equation:

(3) $${r_1}\left( t \right) = x\left( t \right) - \widetilde {\rm IM{F_1}}$$

3. Add white noise in the remainder, which is calculated from Eq. (3) as ${r_1}\left( t \right) + {w_0}{n^j}\left( t \right)$ and decompose it to get second IMF as:

(4) $$\widetilde {\rm IM{F_2}} = \mathop \sum \limits_{j = 1}^m \displaystyle{{\rm IMF_{{\it j}2}^{\it m}} \over m}$$

4. Repeat the steps of (2–3) until the process meets the stoppage criteria, and the remainder/residuals contain only one or two extremes. Finally, the remainder/residual is defined as:

(5) $$R\left( t \right) = x\left( t \right) - \mathop \sum \limits_{k = 1}^K \widetilde {\rm IM{F_{\it k}}}$$

where $k = \; 1,2,..,K$.

After performing CEEMDAN, the next task is to reduce the dimension of multisubsequences (IMFs) (Nava, Matteo & Aste, 2018). To reduce the size of IMFs, excepting the first two IMFs and residuals, the remaining high and low multi-scale components (IMFs) are added with each other respectively as follows:

(6) $$x\left( t \right) = {\rm IMF1} + {\rm IMF2} + \mathop \sum \limits_{J = 1}^s {\rm IM{F_{\it J}}} + \mathop \sum \limits_{k = s + 1}^K {\rm IM{F_{\it k}}} + {\rm Residual}$$

*Prediction step*: In the prediction step, the decomposed IMF components and residual described in Eq. (6) are predicted. For that, two data-driven models (GMDH-NN and its revised form RGMDH-NN) and one traditional statistical model (ARIMA) are used (Nazir et al., 2019). A detailed description of two data-driven and one stochastic models is described in Box (1970) and Ahmadi, Mottaghitalaband & Nariman-Zadeh (2007). A brief description of GMDH-NN is described as follows:

The IMF prediction derived by using GMDH-NN: Except little applications in rivers inflow modeling, GMDH-NN is known for many benefits attached to a wide range of areas. In comparison with ANN and other data-driven models, GMDH-NN, which is a sub-model of ANN, has many advantages: First, GMDH-NN has been proved to be a useful tool for modeling of a complex and non-linear system which is constructed to improve the explicit polynomial model by self-organizing (Ahmadi, Mottaghitalaband & Nariman-Zadeh, 2007). Second, the GMDH-NN is useful in pairwise relationship considerations between all possible selected lagged inputs. All pairs are entered in a neuron to construct output. Further, an evaluation measure is used for neuron selection. The process is continued until the last layer. In the final segment, the only single best-predicted neuron is selected. The only drawback of using GMDH-NN is that it considers the relationship of two inputs while ignores the individual effects of each point. The Architecture GMDH-NN (RGMDH-NN), which is an improved form of GMDH-NN, can be used to overcome this drawback problem, which can utilize two-input relation as well as their individual effects. While the remaining procedure of RGMDH-NN and GMDH-NN is similar. The coefficients of all neurons are estimated with regularized least square estimation method as this method is robust with multicollinearity, which is the characteristic of time series data.

### Copula theory and construction of C-Vine Copula

**Figure 2:**
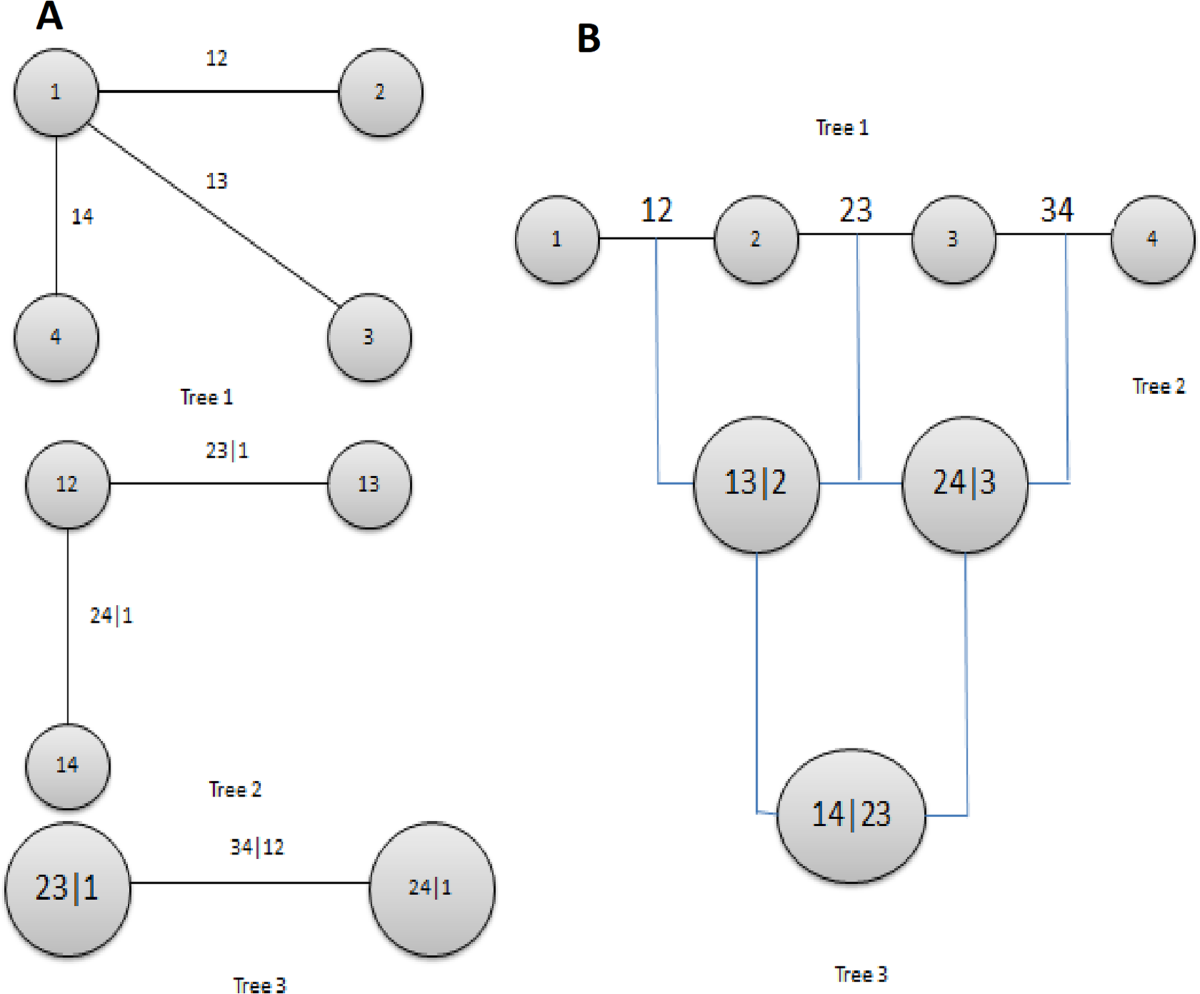
A structure of C-Vine (A) and D-Vine (B) copulas with four variables and three trees. Tree 1 has nodes *N*_1_ = {1, 2, 3, 4} and edges *E*_1_ = {1, 2, 3}, tree 2 has nodes *N*_2_ = {1, 2, 3} and *E*_2_ = {1, 2} and tree 3 has nodes *N*_3_ = {1, 2} and edges *E*_3_ = {1}.

The Copula defined as n-dimensional multivariate distribution function on a unit cube with uniform marginals. An extensive review on Copula includes (Czado et al., 2009; Lee & Salas, 2011; Aghakouchak, 2014; Bedford, Daneshkhah & Wilson, 2016; Bevacqua et al., 2017; Zhao et al., 2017; Yu, Zhang & Singh, 2018). It leads to a suggestion that several researchers agree on using Copula to model the non-linear dependance in applications of finance, hydrology, and climatology. Sklar (1959) proposed a copula by establishing a link between marginal and multivariate distribution as let $F$ be the n-dimensional distribution function and $F({x_i}$) be the marginal distribution of $X = {\left[ {{X_1},{X_2}, \ldots .{X_n}} \right]^T}$, then there exists a copula which defined as:

(7) $$F\left( {{x_1},{x_2}, \ldots .{x_n}} \right) = C\left( {F\left( {{x_1}} \right),F\left( {{x_2}} \right), \ldots F\left( {{x_n}} \right)} \right)$$

(8) $$c(F\left( {{x_1}} \right),F\left( {{x_2}} \right),.,F\left( {{x_n}} \right) = \displaystyle{{d\left( {C\left( {F\left( {{x_1}} \right),F\left( {{x_2}} \right), \ldots F\left( {{x_n}} \right)} \right)} \right)} \over {dF\left( {{x_1}} \right),F\left( {{x_2}} \right), \ldots F\left( {{x_n}} \right)}}$$

where $C$ is a cumulative multivariate distribution function, and $c$ is its density. The readers are advised to look (Nelsen, 2007) for the detailed study of copula theory. A wide variety of symmetrical, that is, Archimedean, elliptical, and asymmetrical copulas are introduced in the literature (Song & Kang, 2011; Almeida, Czado & Manner, 2016; Bevacqua et al., 2017; Wang et al., 2018; Yu, Zhang & Singh, 2018). The most widely used copula functions and their parameters are presented in Tables 1 and 2. Although the Copula is recognized as a powerful tool, it suffers a lack of flexibility when modeling the high dimensional data where complex dependencies exist among the different pairs of variables. Recently, this drawback is covered with the PCCs, also called vines. Specifically, the PCCs are a product of decomposition of bivariate Copula and conditional bivariate copula densities, where all selected bivariate copulas are chosen according to the requirement of their dependance structure (Aas et al., 2009). The initial work on PCCs is found in Kurowicka & Cooke (2006), and later its detail is provided in Yang et al. (2015). Further, it was extended by Bedford, Daneshkhah & Wilson (2016). They explored the case of Gaussian pair copula and called it as R-Vine. They demonstrated that the accurate specification of PCCs makes the multivariate distribution more useful, which is near to reality. The structure of the vine is comprised of connected trees. Different arrangements of vines are available, which is employed according to the requirement as R-Vine, C-Vine and D-Vine. The schematic view of C-Vine and D-Vine is presented in Fig. 2. Here in our study, the C-Vine copula structure is used to model the joint dependance structure of multi-site rivers. The four-dimensional C-Vine copula density is expressed as follows (Allen, McAleer & Singh, 2017) for four variables:

(9) $$f\left( {{x_1},..,{x_4}} \right) = \prod\limits_{i = 1}^4 f \left( {{x_i}} \right)\prod\limits_{k = 1}^3 {\prod\limits_{l = 1}^{4 - k} {{c_{k,k + l|1,..,k - 1}}} } \left( {F\left( {{x_k}|{x_1}, \ldots ,{x_{k - 1}}} \right),F\left( {{x_{k + l}}|{x_1}, \ldots ,{x_{k - 1}}} \right),F\left( {{x_1}, \ldots ,{x_{k - 1}}} \right)} \right)$$

where index $k$ identifies trees and $i$ defines edges of all trees, ${c_{k,k + 1|1,..,k - 1}}$ works according to its subscript and $F\left( {{x_{}}|v} \right)$ for $m$ dimensional vector $v$ presents the conditional distribution function, which is calculated as (Allen, McAleer & Singh, 2017):

(10) $$F\left( {x|v} \right) = \displaystyle{{d({c_{xv|{v_{ - j}}}}\left( {F\left( {x|{v_{ - j}}} \right),F\left( {{v_j}|{v_{ - j}}} \right)} \right)} \over {dF\left( {{v_j}|{v_{ - j}}} \right)}}$$

where ${v_j}$ is an arbitrary component of $v$ and ${v_{ - j}}$ denotes the $\left( {m - 1} \right)$ dimensionl vector excluding ${v_j}$.

**Table 1:** Archimedean and elliptical copula family with corresponding generator, inverse function, Kendall τ and its range.

Copula	${\rm{\phi}}\left({u} \right)$	${C}\left( {{u},{v}} \right)$	Kendall’s τ	Parameter Range
Clayton	$\displaystyle{{{t^{ - \rm \theta }} - 1} \over \rm \theta }$	$C{\left( {{u^{ - \rm \theta }} + {v^{ - \rm \theta }} - 1} \right)^{ - 1/\rm \theta }}$	$\displaystyle{{\rm \theta } \over {{\rm \theta } + 2}}$	${\rm \theta } > \left( {0,\infty } \right)$
Gumbel	${\left( { - lnt} \right)^{\rm \theta} }$	${\rm exp}( - {\left[ { - ln{u^{\rm \theta} } - ln{v^{\rm \theta} }} \right]^{\textstyle{1 \over {\rm \theta} }}}$	$\displaystyle{{{\rm \theta } - 1} \over {\rm \theta }}$	${\rm \theta} > 1$
Frank	-ln$\left( {\displaystyle{{{e^{ - {\rm \theta} t}} - 1} \over {{e^{ - {\rm \theta} }} - 1}}} \right)$	$- \displaystyle{1 \over {\rm \theta} }\left( {1 + \displaystyle{{({e^{ - {\rm \theta} u}} - 1)\left( {{e^{ - {\rm \theta} v}} - 1} \right)} \over {{e^{ - {\rm \theta} }} - 1}}} \right)$	$1 - \displaystyle{4 \over {\rm \theta} }\left[ {1 - {D_1}{{\left( {\rm \theta} \right)}^*}} \right]$	${\rm \theta }$> (−∞, ∞)
Gaussian		${\rm \phi _\rho }\left( {{\rm \phi}_{{u_1}}^{ - 1},{\rm \phi} _{{u_2}}^{ - 1}} \right)$	$\displaystyle{2 \over \pi }{\rm arcsin}\left( \rm \rho \right)$	ρ > (−1, 1)
Student-t		${t_{\rho ,\upsilon }}\left( {{t_\upsilon }_{{u_1}}^{ - 1},{t_\upsilon }_{{u_2}}^{ - 1}} \right)$	$\displaystyle{2 \over \pi }{\rm arcsin}\left( \rm \rho \right)$	ρ > (−1, 1), ν > 2

**Table 2:** BB family of Copula with their generator, Kendall’s tau and parameter space.

Copula	${\bf{\rm{\phi}}}\left( \bf{u} \right)$	τ	Parameter range
(BB6)	${\left( { - {\rm log}\left[ {1 - \; {{\left( {1 - t} \right)}^{\rm \theta} }} \right]} \right)^{\rm \delta} }$	$1 + \displaystyle{4 \over {\rm \delta \rm \theta }}\mathop \int \nolimits_0^1 ( - {\rm log}(1 - \; {\left( {1 - t} \right)^{\rm \theta} })$	${\rm \theta} \ge 1$
		$*\left( {1 - t} \right)\left( {1 - {{\left( {1 - t} \right)}^{\rm \theta}}} \right)))dt$	${\rm \delta} \ge 1$
(BB7)	${\left( {1 - {{\left( {1 - t} \right)}^{\rm \theta} }} \right)^{ - {\rm \delta} }} - 1$	$1 + \displaystyle{4 \over {{\rm \theta} {\rm \delta} }}\mathop {\rm \oint }\nolimits_0^1 \matrix{ {( - {{\left( {1 - {{\left( {1 - t} \right)}^{\rm \theta} }} \right)}^{{\rm \delta} + 1}}} \cr {*\displaystyle{{{{\left( {1 - {{\left( {1 - t} \right)}^{\rm \theta} }} \right)}^{ - {\rm \delta} }} - 1} \over {{{\left( {1 - t} \right)}^{{\rm \delta} - 1}}}}dt} \cr }$	${\rm \theta} \ge 1$
			${\rm \delta} \ge 1$
(BB8)	$- {\rm log}\left[ {\displaystyle{{1 - {{\left( {1 - {\rm \delta} t} \right)}^{\rm \theta} }} \over {1 - {{\left( {1 - {\rm \delta} } \right)}^{\rm \theta} }}}} \right]$	$1 + {4 \over {\theta \delta }}\mathop \oint \limits_0^1 \left( { - \log \left[ {{{(1 - \delta t)}^{\theta - 1}}{{(1 - \delta )}^\theta } - 1} \right](1 - t\delta )(1 - {{(1 - t\delta )}^{ - \theta }})} \right)dt$	${\rm \theta} \ge 1$
			${\rm \delta} \in [0, 1]$

#### Choosing pair copula families and estimation of parameters

There are several pair-copula families, that is, Frank, Gumbel, Clayton, Gaussian and *t* as listed in Tables 1 and 2. The copula pair is typically chosen in each tree one by one according to different model selection criteria like Akaike Information Criterion (AIC), the Bayesian Information Criterion (BIC), and goodness-of-fit test criteria. Aas et al. (2009) used AIC and BIC for the selection of bivariate pair of Copula for vine structure. However, care should be taken in selecting the bivariate copulas as the selection of a copula families in the tree for vine structure depends on the choice on the introductory level copulas due to the connected trees. In this study, the AIC, BIC (Mirbagherijam et al., 2015), and maximum log-likelihood methods are used as the most reliable selection criterion to select the possible pair of copulas and its conditionals. For the parameter estimation, the maximum likelihood method is employed for each couple of copulas and its conditional.

#### Marginal distribution

To proceed with Copula, the standard uniform distribution is required, which is the inverse transformation of the marginal distribution. According to previous studies, both parametric and non-parametric distributions are used Chen & Guo (2019) to get appropriate marginal distribution. To fit the first-stage residuals resulted from CEEMDAN-R-MM, Empirical, normal, and *t* distribution functions are used. The detailed description of normal, *t* and empirical distribution function is given in Chen & Guo (2019). The parameters of the marginal distribution are estimated through the maximum likelihood method and to verify the best fit distribution, Kolmogorov–Smirnov (K–S) test is used.

#### Simulation from C-Vine based conditional distribution

The simulation algorithm of C-Vine is defined as follows:

First four independent standard uniform random variables $\left( {{t_1},{t_2},{t_3},{t_4}} \right)$ are generated. Then these values are used as probability levels to determine (${u_1},{u_2},{u_3},{u_4})$ through the following equations:

(11) $${u_1} = {t_1}$$

(12) $${u_2} = F({t_2}|{u_1})$$

(13) $${u_3} = F({t_3}|{u_1},{u_2})$$

(14) $${u_4} = F({u_4}|{u_1},{u_2},{u_3})$$

where the conditional distributional function is calculated through Eq. (10). Once ${u_i}$’s where *i* = 1, 2, 3, 4 are simulated, the corresponding ${x_i}$’s are calculated from the inverse normal CDFs.

### Comparison of proposed model with benchmark models

The proposed model is compared with three benchmark models described as following:

1. First, the Vector Autoregressive (VAR) model of Ledolter (1978) is considered where the dependance structure of multi-site rivers inflow is modeled directly without assuming their multi-scale and joint probabilistic structure.
2. Second, the copula-based ARMA model of Singh et al. (2018) is selected for the comparison of the proposed model. The copula-based ARMA model only considers the joint dependance structure of multi-site rivers inflow without assuming the multi-scale characteristics into account.
3. Third, CEEMDAN-R-MM, work of Nazir et al. (2019) is considered where only multi-scale characteristics are modeled without considering the joint dependance structure among multi-site river inflow.

### Evaluation criteria

The prediction accuracy of the proposed model is evaluated using four evaluation measures such as MAD, Mean Absolute Relative Error (MARE), Nash-Sutcliffe Efficiency (NSE) and Mean Square Error (MSE) are used to observe the prediction performance. The following are their Eq.’s from (15–18), respectively:

(15) $${\rm MAD} = \displaystyle{1 \over N}\mathop \sum \limits_{t = 1}^N \left| {x\left( t \right) - \hat x\left( t \right)} \right|$$

(16) $${\rm MARE} = \displaystyle{{100\% } \over N}\mathop \sum \limits_{t = 1}^N \left| {\displaystyle{{x\left( t \right) - \hat x\left( t \right)} \over {x\left( t \right)}}} \right|$$

(17) $${\rm NSE} = 1 - \displaystyle{{\mathop \sum \limits_{t = 1}^N {{\left( {\hat x\left( t \right) - x\left( t \right)} \right)}^2}} \over {\mathop \sum \limits_{t = 1}^N {{\left( {x\left( t \right) - \underline{x} \left( t \right)} \right)}^2}}}$$

and

(18) $${\rm MSE} = \displaystyle{1 \over N}\mathop \sum \limits_{t = 1}^N {\left( {x\left( t \right) - \hat x\left( t \right)} \right)^2}$$

Where $x\left( t \right)$ is the original series of data and $\hat x\left( t \right)$ is the predicted series of data.

## Case Study and Experimental Design

Being the most substantial irrigation, insensible water resource system, and source of power generation in Pakistan, Indus River Basin (IRB) is considered for the application of the proposed methodology. The time-series data for the four rivers (River Indus, the River Jhelum, the River Chenab and the River Kabul) contributing significantly to the water system of IRB is utilized to validate the proposed methodology. These streams are generating severe flooding due to melting snow or glacier, and torrential monsoon precipitation in Pakistan. The 13% mountainous regions of the Upper Indus Basin (UIB) cover 13,680 km^2^ area of the glacier in Pakistan, which is significantly contributing to the IRB system. Pakistan suffered floods, almost one wave every three years from 1950 to 2011. Ali (2013) reported that $19 billion economic losses have occurred, total 109, 822 villages have damaged, and a total of 8,887 people have died due to floods in Pakistan. The only flood that occurred in 2010 caused $10 billion, the highest financial loss of Pakistan. Therefore, to assess the proposed model, it is appropriate to use rivers data of IRB as an illustrative case study.

**Figure 3:**
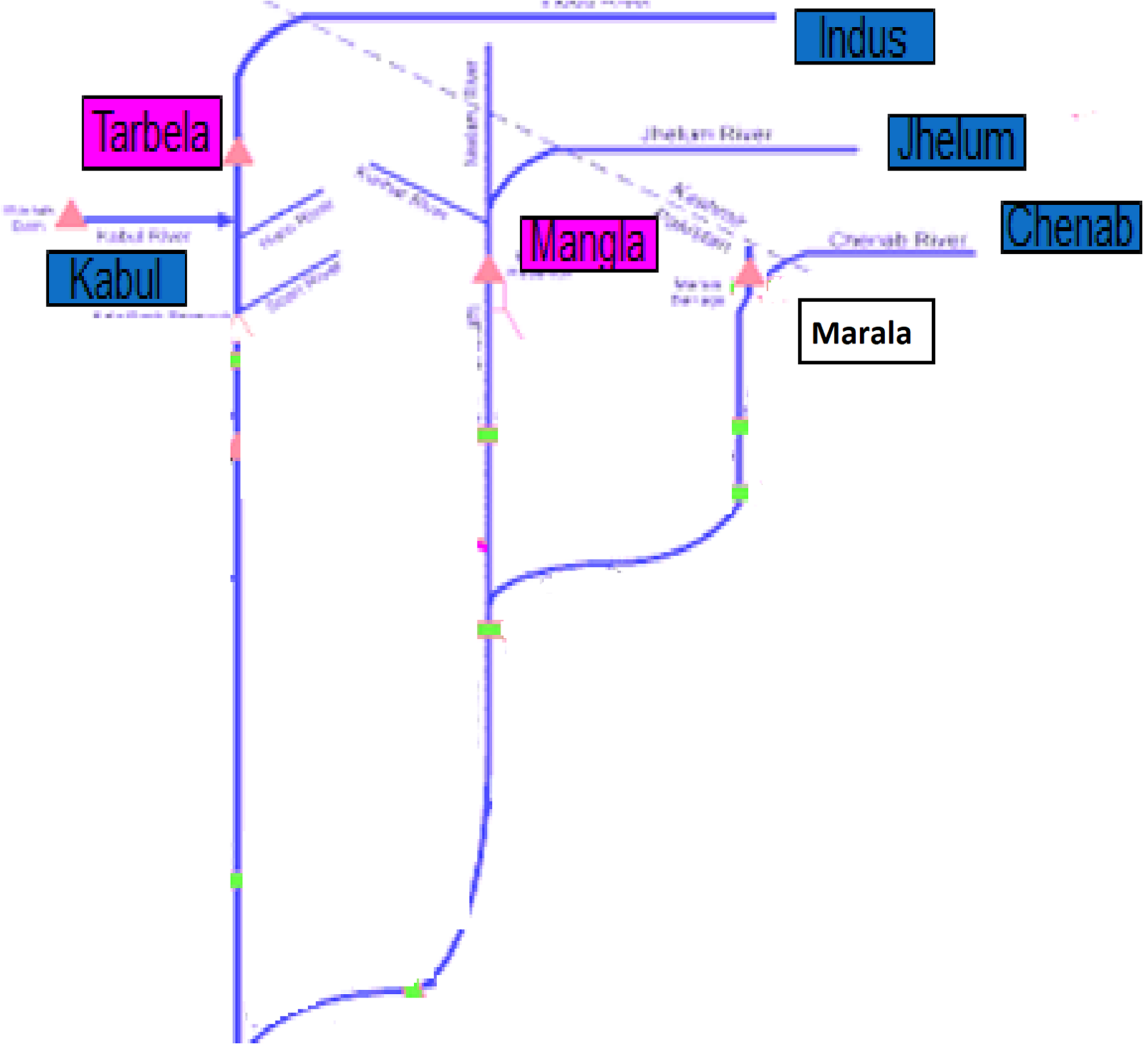
Network of selected rivers: Indus, Jhelum, Chenab and Kabul.

The daily river inflow data set used in this study is comprised on 1st January 2015 to 19 November 2018. For the application of the proposed objective, the daily inflow of the Indus River at Tarbela with its two principal left and one right bank tributaries: Jhelum River at Mangla, Chenab River at Marala and Kabul River at Nowshera respectively are selected. The schematic view of rivers chosen is presented in Fig. 3. The daily inflow data is measured in 1000 CUSECS, which was acquired from the site of Pakistan Water and Power Development Authority (WAPDA).

## Results

**Figure 4:**
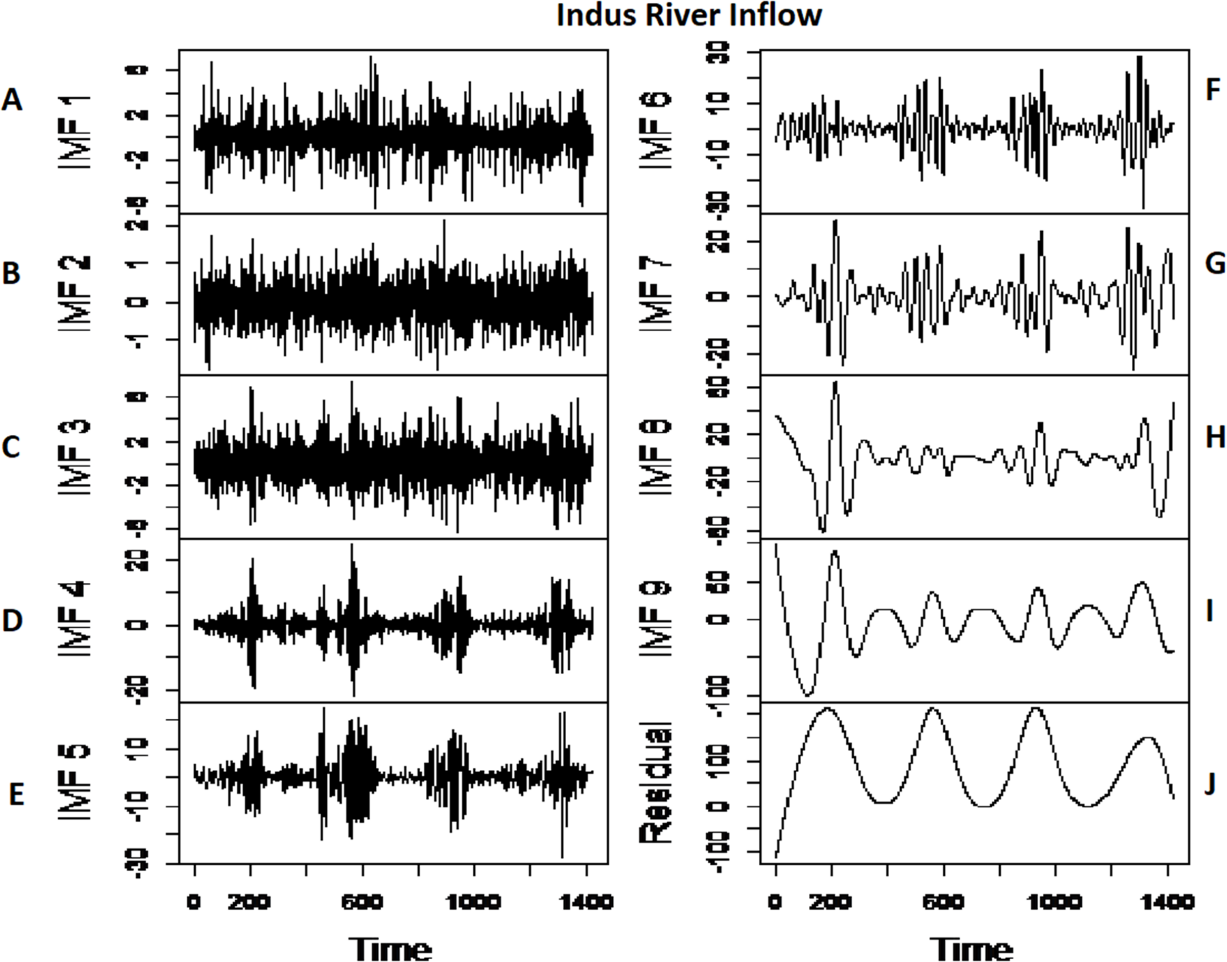
The CEEMDAN based decomposition of Indus rivers inflow where (A–E) first five IMFs of Indus river inflow and (F–J) remaining IMFs of Indus river inflow.

**Figure 5:**
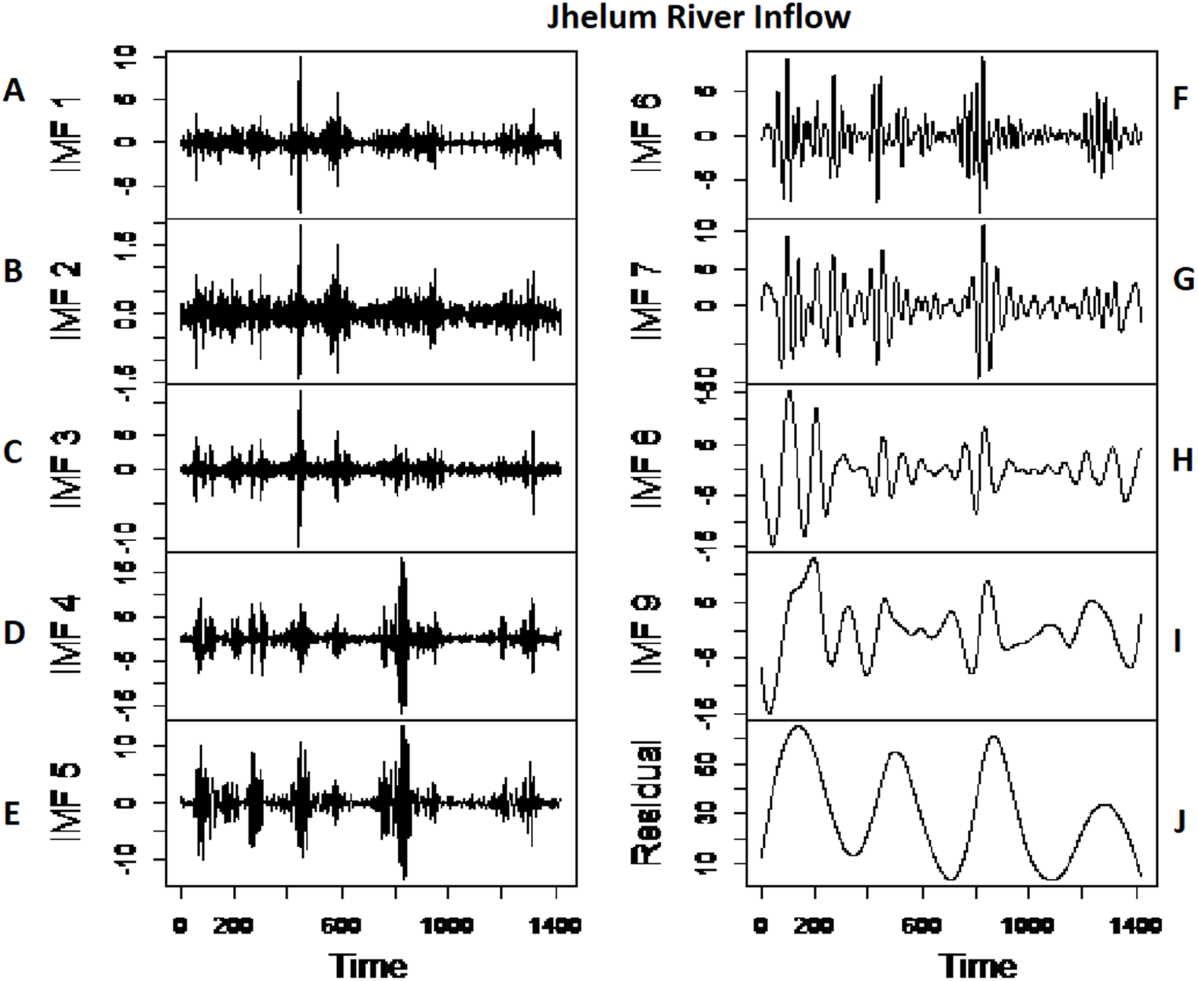
The CEEMDAN based decomposition of Jhelum river inflow where (A–E) First five IMFs of Jhelum river inflow and (F–J) remaining IMFs of Jhelum river inflow.

*The first-stage proposed model results:* the first stage of the proposed strategy is comprised of modeling the multi-scale features of each river inflow data to proceed with the multi-site joint dependance. The proposed approach is applied to the daily inflow data set of four rivers, that is, Indus, Kabul, Jhelum and Chenab. The river inflow data for all case studies is first decomposed into multi-scale IMFs through CEEMDAN. All four river’s inflow data is decomposed into nine IMFs and one residual (Nazir et al., 2019) as shown in Figs. 4 and 5 for Indus river inflow and Jhelum river inflow respectively. The white noise amplitude is set as 0.2 Di, Yang & Wang (2014), and a maximum number of ensemble members are selected as 1000. The dimension of extracted CEEMDAN based nine IMFs is further reduced (CEEMDAN-R) to save time and labor of modeling each IMF individually. To obtain CEEMDAN-R, except for the first two IMFs and the last residual, the remaining IMFs showing the same high and low multi-scale components are added with each other, respectively. The first two IMFs are predicted alone as they both shown complex and the highest frequency, as depicted in Figs. 4 and 5, for Indus and Jhelum river, inflow data respectively. From the remaining seven IMFs, the first four IMFs and last three IMFs are added separately with each other as they showed the same high and low multi-scale components. To predict the IMF1, IMF2, added high and low multi-scale elements and residuals, GMDH, RGMDH and ARIMA models are applied, and the best one method with minimum MDE, MARE, and MSE is selected among all prediction methods. For prediction purpose, data is divided into 80% for training and while the second is 20% for testing. The training results of the first stage proposed model CEEMDAN-R-MM for all case studies are presented in Table 3. The residuals from this stage model, CEEMDAN-R-MM, are used as inputs in the second-stage model to get improved and final multi-site rivers inflow prediction.

**Table 3:** Results of the proposed model (C-Vine based CEEMDAN-R-MM) and benchmark (VAR, ARIMA-COP and CEEMDAN-R-MM models).

Rivers inflow	Model	MAD	NSE	MARE	MSE
Indus inflow	VAR	4.9069	0.9899	0.0715	77.9964
Jhelum inflow		3.7185	0.9042	0.0715	50.5069
Chenab inflow		2.7334	0.9885	0.0976	28.4530
Kabul inflow		4.6269	0.8849	0.1944	99.9116
Indus inflow	ARIMA-COP	4.3562	0.9915	0.0744	64.4888
Jhelum inflow		3.6253	0.9158	0.1358	46.7833
Chenab inflow		2.6468	0.9608	0.1043	24.9336
Kabul inflow		4.7105	0.8844	0.1429	100.271
Indus inflow	CEEMDAN-R-MM	2.2145	0.9989	0.0652	8.0779
Kabul inflow		1.2822	0.9967	0.0730	2.8825
Chenab inflow		0.8694	0.9980	0.0576	1.2689
Jhelum inflow		0.8664	0.9978	0.0454	1.2971
Indus inflow	C-Vine based CEEMDAN-R-MM	2.1771	0.9990	0.0770	7.8407
Kabul inflow		0.9195	0.9978	0.0687	1.3767
Chenab inflow		1.2826	0.9966	0.1458	2.8982
Jhelum inflow		0.8985	0.9976	0.0691	1.4090

**Figure 6:**
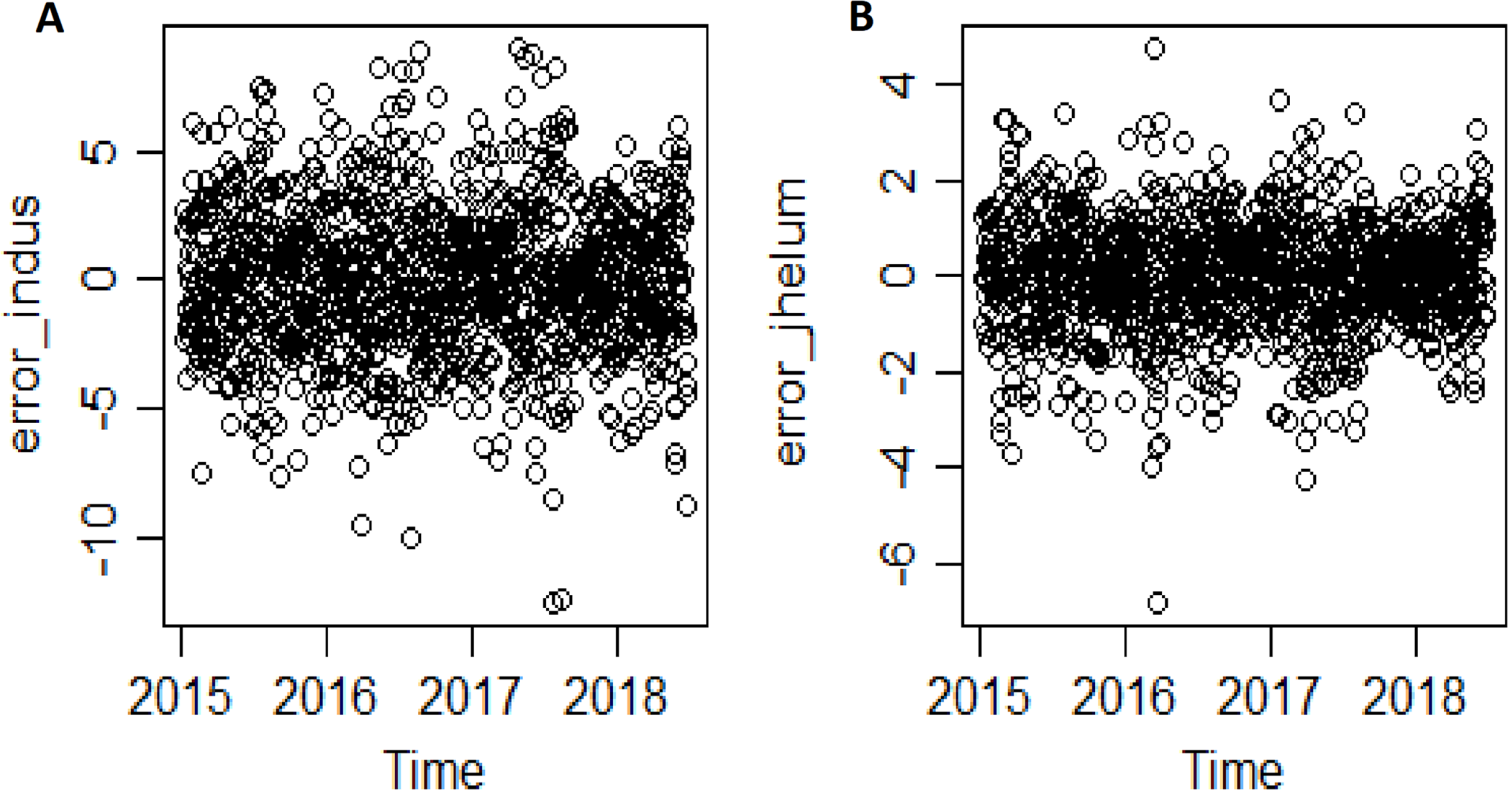
Residuals from the first-stage CEEMDAN-R-MM method for (A) Indus and (B) Jhelum river inflow.

**Figure 7:**
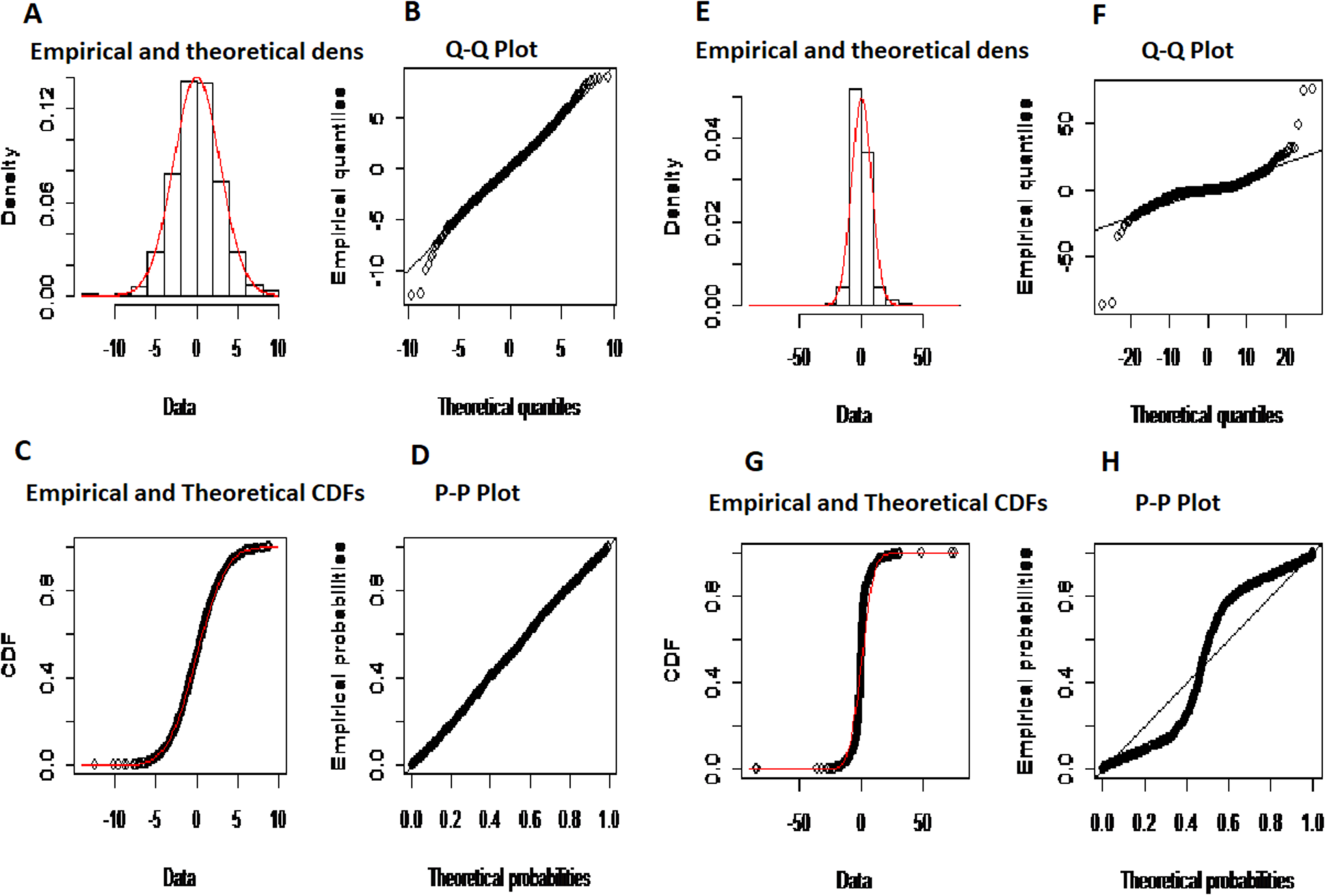
The empirical (red and black dotted line) and theoretical (straight black line) normal distribution of errors resulting from CEEMDAN-R-MM (A–D) and (right) ARMA method (E–H).

**Figure 8:**
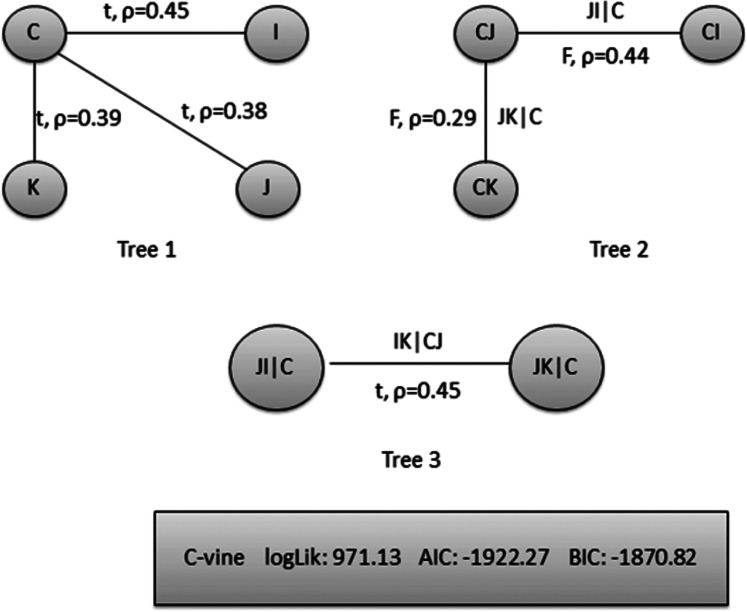
Structure of pair copula decomposition of 4-D C-Vine copula for Indus and Kabul rivers inflow simulation conditioned on Jhelum and Chenab rivers inflow where C is showing Chenab, J for Jhelum, K for Kabul and I for Indus river inflow with its AIC, BIC and log-likelihood values.

**Figure 9:**
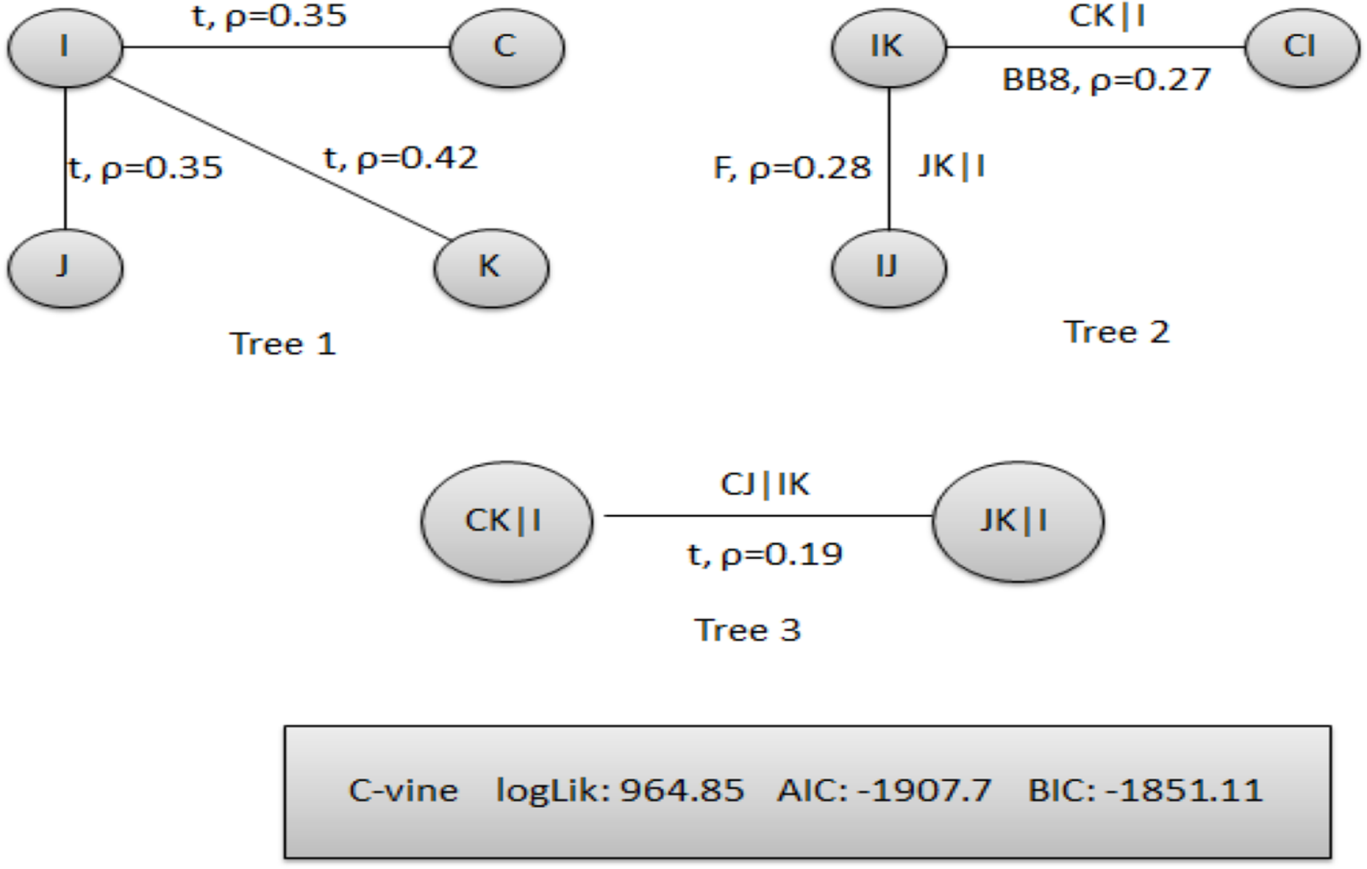
Structure of pair copula decomposition of 4-D C-Vine copula for Jhelum and Chenab rivers inflow simulation conditioned on Indus and Kabul rivers inflow where C is showing Chenab, J for Jhelum, K for Kabul and I for Indus river inflow with its AIC, BIC and log-likelihood values.

**Figure 10:**
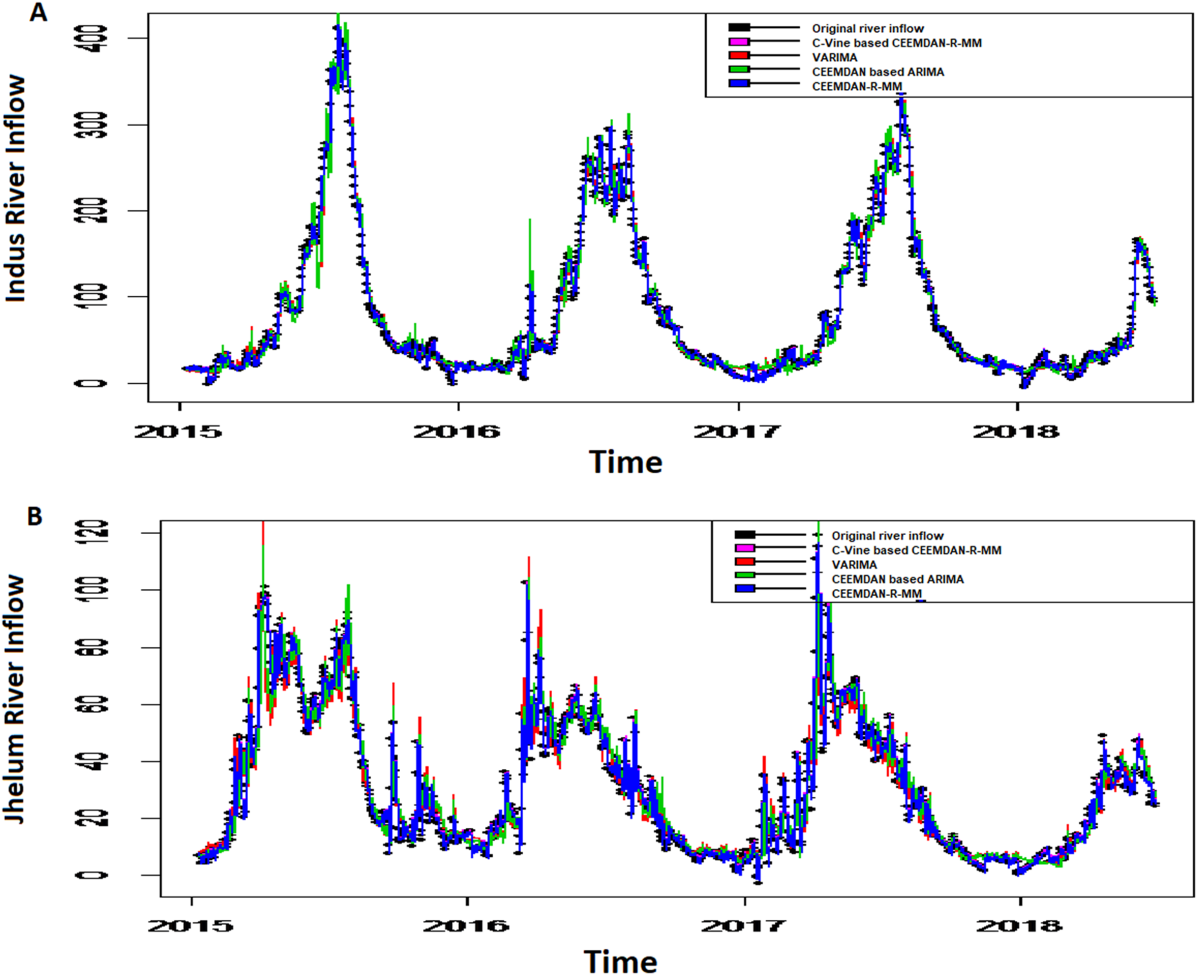
The predicted graph for all case studies of proposed two-stage C-Vine based CEEMDAN-R-MM model with the predicted values of benchmark models (VAR, Copula based ARMA, first-stage CEEMDAN-R-MM) for (A) Indus river inflow, (B) Jhelum river inflow.

**Figure 11:**
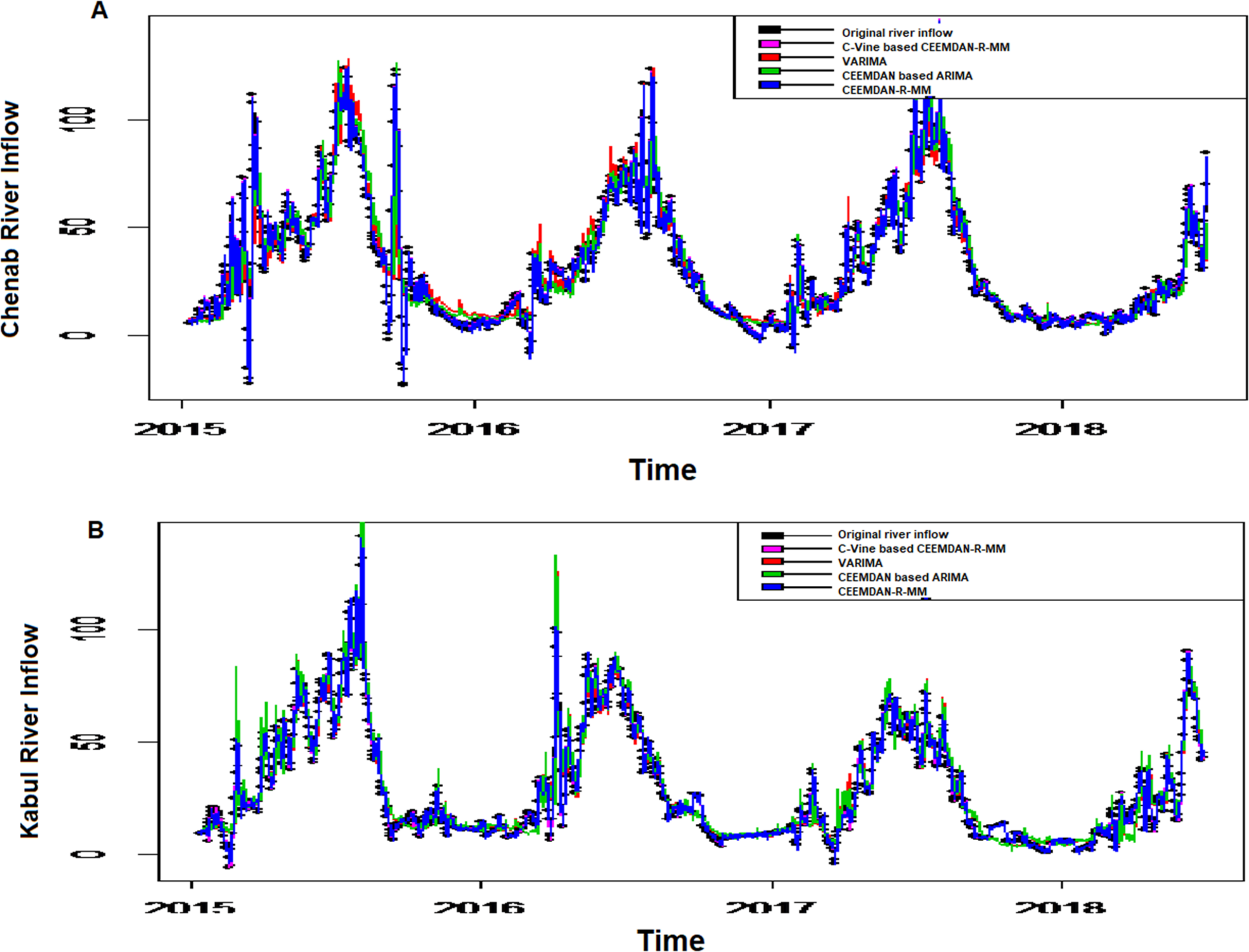
The predicted graph for all case studies of proposed two-stage C-Vine based CEEMDAN-R-MM model with the predicted values of benchmark models (VAR, Copula based ARMA, first-stage CEEMDAN-R-MM) for (A) Chenab river inflow, (B) Kabul river inflow.

*The second-stage proposed C-Vine based CEEMDAN-R-MM model results:* to model the mutual dependance of multi-site river inflow data, the residuals from the first stage proposed model, CEEMDAN-R-MM is used. The accuracy of the first stage model is evaluated through the estimated residuals generated from CEEMDAN-R-MM by using modified Q-statistic Ljung & Box (1978) and the Lagrange multiplier Evans & Patterson (1985) tests, which are used to check the serial correlation of estimated residuals. Both modified Q-statistics and Lagrange multiplier revealed that there is no autocorrelation in the estimated residuals for all case studies at the 5% level. The independent residuals from the first stage CEEMDAN-R-MM model for the Indus and Jhelum rivers inflow are shown in Fig. 6. Before applying the C-Vine method, the correlation of residuals of multi-site river inflow data needs to be estimated. For that reason, Kendall’s rank correlation measure is used. The estimated values of Kendall’s rank correlation are given in Table 4. From Table 4, it is depicted that there exists a correlation between all pairs of multi-site river inflow data except for Jhelum and Chenab rivers inflow. Further, to proceed with C-Vine Copula, cumulative distribution functions are fitted using empirical, normal, and t-distribution functions. The appropriate distribution is verified according to the *p*-value of the K–S test. The null hypothesis of the K-S test is that the residuals follow a specified distribution here, we set normal and t distribution. The *p*-value 0.200 against normal distribution, confirms that all residuals are determined appropriate because it is more significant than others distribution see Fig. 7. The advantage of the proposed first-stage model CEEMDAN-R-MM has also confirmed with the normal distribution that the errors confirm the assumption of IID as it can be seen from Fig. 7. To increase the prediction precision of river inflow data, this joint dependance structure of multi-site rivers inflow is incorporated with C-Vine Copula. Different bivariate copula functions, as listed in Tables 1 and 2, are fitted to make the building block between pairs of rivers simultaneously of C-Vine Copula. The most appropriate fitted bivariate and conditional bivariate copula functions are selected based on the maximum log-likelihood, lower value of AIC and BIC. The selected C-Vine conditional structure with AIC and BIC values for the Indus and the Kabul is shown in Fig. 8, whereas for the Chenab and Jhelum it is shown in Fig. 9. For Jhelum and Chenab, it is cleared that the correlation is low for all pairs, as depicted in Fig. 9. The simulation for each river’s inflow data is done using Eqs. (11)–(13). Finally, the simulated values are added in the predicted values of the first stage (the CEEMDAN-R-MM model) to get final values for all four case studies. The overall performance of the proposed model, C-Vine based CEEMDAN-R-MM, are compared with benchmark models (VAR by Ledolter (1978), Copula-based ARMA Singh et al., 2018, and the first-stage CEEMDAN-R-MM (Nazir et al., 2019)) for all four case studies. The results of proposed and benchmark models are given in Table 3. The predicted river inflow data for Indus and Jhelum river inflow for proposed and benchmark models is depicted in Fig. 10 and for Kabul and Chenab is depicted in Fig. 11. From Table 3, it can be observed that the proposed two-stage C-Vine based CEEMDAN-R-MM model outperforms the other benchmark models for Indus and Kabul river inflow as shown in Table 3 with bold measure values. On the other hand, the rivers as Jhelum and Chenab showing insignificant relationships are better modeled only through the first-stage proposed model (CEEMDAN-R-MM) as compared to the two-stage proposed model and other benchmark models as indicated in Table 3.

**Table 4:** Estimated values of Kendall’s correlation.

Rivers	Indus	Jhelum	Chenab	Kabul
Indus	1.0000	0.4410	0.5441	0.7468
Jhelum		1.0000	0.6794	0.5844
Chenab			1.0000	0.6207
Kabul				1.0000

## Discussion

The following discussion is inferred based on the training error presented in Table 3:

*Overall comparison of the first stage and second stage proposed model:* the overall performance of the proposed two-stage C-Vine based CEEMDAN-R-MM model shows prediction improvement on all other three methods listed in Table 3 MAD, NSE, MARE and MSE for the Indus and Kabul rivers inflow. However, for the Jhelum and the Chenab rivers inflow as they did not provide any significant correlation among pairs of variables as depicted in Fig. 9, only the first-stage model (CEEMDAN-R-MM; Nazir et al., 2019), provides satisfactory results for Jhelum and Chenab rivers inflow than all other existing work of Ledolter (1978) and Singh et al. (2018) and two-stage novel C-Vine based CEEMDAN-R-MM model. It can be observed from our results that by utilizing important information that is present in data, one can enhance the quality of complex hydrological time series data.

*Comparison of existing benchmark models:* for all four case studies, it can be observed from Table 4 that VAR performs poorly as compared with CEEMDAN-R-MM and copula-based ARIMA model as it does not consider the multi-scale characteristics of time-varying and non-linear data. Moreover, by combining Copula with ARMA, the prediction performance of multi-site rivers inflow is increased over a simple VAR model, as shown in Table 3 for all four case studies.

Overall it is concluded that for the significant correlation among rivers, our proposed C-Vine based CEEMDAN-R-MM and for the non-significant association between rivers, our first-stage proposed model CEEMDAN-R-MM performs well over the works of Ledolter (1978) and Singh et al. (2018). It is concluded that the performance of multi-site river inflow data can enhance by providing the maximum information which exists between complex multivariate time series data.

## Conclusion

Prediction of multi-site river inflow has become a hot topic for hydrological researchers today. In this study, the IRB of Pakistan has been selected for predicting the multi-site river inflow by using its four main rivers: Indus, Kabul, Jhelum and Chenab. A novel C-Vine based CEEMDAN-R-MM model is proposed to predict such multi-site rivers inflow that considered its complex dependance structure. We found that the accuracy of prediction can be improved by appropriately modeling the dependance structure of the multi-site river inflow data.

### Further recommendations

In this paper, we proposed a C-Vine based CEEMDAN-R-MM method to model multi-site river inflow data, which proved fruitful over simple single-site river inflow modeling by utilizing the dependance structure which exists between rivers. However, it is also seen that when there is no dependance between river inflow data, only the CEEMDAN-R-MM model provides efficient results. Overall, these conclusions are applied to the river system studied and may be used for river systems with similar flow characteristics.

## Supplemental Information

10.7717/peerj.10285/supp-1 Supplemental Information 1 River inflow data used to validate proposed methods.

## Additional Information and Declarations

### Data Availability

The following information was supplied regarding data availability: Data used in analysis is available as a Supplemental File.
